# Deep reinforcement learning control unlocks enhanced heat transfer in turbulent convection

**DOI:** 10.1073/pnas.2506351122

**Published:** 2025-09-09

**Authors:** Zisong Zhou, Xiaojue Zhu

**Affiliations:** ^a^Max Planck Institute for Solar System Research, Göttingen 37077, Germany

**Keywords:** turbulent convection, reinforcement learning, turbulence control

## Abstract

Turbulent convection governs heat transport in both natural and industrial settings, yet optimizing it under extreme conditions remains a significant challenge. Traditional control strategies, such as predefined temperature modulation, struggle to achieve substantial enhancement. Here, we introduce a deep reinforcement learning (DRL) framework that autonomously discovers optimal control policies to maximize heat transfer in turbulent Rayleigh-Bénard convection. By dynamically adjusting wall temperature fluctuations, the DRL agent achieves a heat transfer enhancement of up to 38.5%, exceeding the 20 to 25% limit of conventional methods. The learned strategy reveals a nonlinear state–action relationship, inducing a fully modulated boundary layer regime. Furthermore, we distill the DRL insights into a simplified bang-bang control model, which retains comparable performance (up to 40.0% enhancement) and, crucially, generalizes to unseen, higher Rayleigh number cases without additional training. Our results demonstrate the power of machine learning in turbulence control and reveal a framework with potential for intelligent heat transfer optimization in real-world applications.

## Significance

Enhancing heat transfer in turbulent flows is vital for energy systems and industrial processes, yet conventional methods yield limited gains. We demonstrate how artificial intelligence autonomously discovers superior control strategies. A deep learning system dynamically adjusts thermal boundaries in turbulence simulations, achieving 38.5% heat transfer enhancement—over 50% better than traditional approaches. The AI-derived strategy is distilled into a simple formula that retains effectiveness even in extreme, unencountered conditions without additional computations. This breakthrough contributes to bridging the gap between data-driven control and real-world applications, offering a framework for advanced turbulent flow control.

Turbulent convection plays a fundamental role in shaping both natural phenomena and engineered systems, driving energy transport across a vast range of scales. In nature, buoyancy-driven turbulent convection governs atmospheric dynamics, influencing weather patterns and global climate systems (1), while also sustaining ocean currents that regulate thermohaline circulation (2). It underlies key geophysical processes, such as mantle convection that shapes Earth’s interior (3), and astrophysical phenomena, including stellar energy transport and magnetic field generation (4, 5). In technology, turbulent convection is central to optimizing heat exchangers (6), enhancing thermal management in electronic cooling, and improving industrial processes such as semiconductor crystal growth (7) and advanced thermal stabilization (8). Despite its ubiquity, turbulent convection remains a challenge to control and predict, motivating decades of research into its fundamental mechanisms.

An archetypal model for investigating turbulent convection is Rayleigh-Bénard (RB) convection, where a horizontal fluid layer of height H is uniformly heated from below at temperature $T_{b}$ and cooled from above at temperature $T_{t}$, resulting in a temperature difference $T_{b}-T_{t}=\Delta T$. This simplified system serves as a foundational framework for exploring convection and its relevance to both natural phenomena and engineering applications. The heat transfer properties of RB convection are defined by three key dimensionless numbers: the Rayleigh number (Ra), the Prandtl number (Pr), and the Nusselt number (Nu) (4, 5, 9, 10). Ra quantifies the intensity of thermal driving and is expressed as $Ra=\alpha g\Delta TH^{3}/\left(\nu \kappa \right)$, where α is the thermal expansion coefficient, g the gravitational acceleration, ν the kinematic viscosity, and κ the thermal diffusivity. Meanwhile, Pr characterizes the fluid’s momentum-to-thermal diffusivity ratio through Pr=ν/κ. In contrast, the Nusselt number (Nu) measures the efficiency of heat transfer relative to pure conduction, defined as Nu=Q/(λΔT/H), where Q is the total heat flux and λ is the thermal conductivity. Nu reflects the enhancement of heat transport due to convection and serves as a critical metric for evaluating thermal performance in turbulent flows.

In recent years, various strategies have been employed to control heat transfer in RB convection, with a particular focus on modifying flow structures and boundary layers. These approaches include surface roughness (11, 12), time-varying heating (13), spatial temperature modulation (8, 14), and the application of external forces such as vibrations (7), magnetic fields (15), and the addition of particles (16). For example, spatially harmonic heating has been shown to affect the emission of thermal plumes and the large-scale circulation, thereby enhancing heat transfer by modulating convective dynamics (14). Low-wavenumber wall temperature fluctuations, with a sinusoidal distribution along the horizontal direction, can notably increase Nu, with a maximum heat transfer enhancement of approximately 25% observed in three-dimensional simulations (14). However, despite these advances, existing methods often rely on predefined control strategies, which limit their optimization potential. It remains unclear whether current control strategies can be improved to achieve greater heat transfer enhancement, highlighting the need for further investigation.

To address the optimization challenges in flow control techniques for RB convection, the application of DRL emerges as a promising solution. DRL has garnered significant attention in various domains, including video classification (17) and speech recognition (18), due to its ability to autonomously optimize complex decision-making tasks. In fluid mechanics, DRL is increasingly utilized for flow control problems (19–33), offering an innovative shift from conventional methods that often rely heavily on human insights. By leveraging neural networks, DRL can build nonlinear models between inputs and controls, potentially uncovering adaptive strategies that are more finely attuned to the complex dynamics of turbulence (34, 35), potentially leading to substantial improvements in control strategy performance. While DRL has shown promise in stabilizing flow and convection patterns at relatively low Ra (22, 29, 33), controlling RB convection at high Ra (not lower than $10^{7}$), where turbulence becomes pronounced (36), remains a significant challenge. This high-Ra regime is more commonly encountered in engineering applications, where efficient heat transfer enhancement is crucial.

In this work, we deploy DRL method to optimize heat transfer in high-Ra RB convection. While existing strategies rely on predefined actuation patterns, our approach enables autonomous discovery of nonlinear control policies that dynamically adapt to turbulent convection. We demonstrate that DRL-driven wall temperature modulation achieves heat transfer enhancement up to 38.5% at $Ra=5\times 10^{8}$ for Pr=1, significantly outperforming traditional methods. This improvement is attributed to the emergence of a fully modulated boundary layer regime, where thermal perturbations penetrate deeply into the central flow region. Notably, the learned strategy can be distilled into a simplified threshold-based model without training process, retaining comparable performance while reducing computational complexity, which even achieves 40.0% heat transfer enhancement under higher $Ra=1\times 10^{9}$.

## Results

### DRL-Based Control Strategies.

In this study, heat transfer enhancement is achieved through the implementation of nonuniform heating on the lower wall. During the control process, the average temperature $T_{b}$ of the lower wall x=0 remains constant, while spatially and temporally varying temperature fluctuations $T_{w}^{\prime }\left(y,z,t\right)$ serve as control inputs. Here, (x,y,z) represents the coordinates in the wall-normal direction and the two orthogonal horizontal directions, respectively, with t denoting time.

**Fig. 1.**
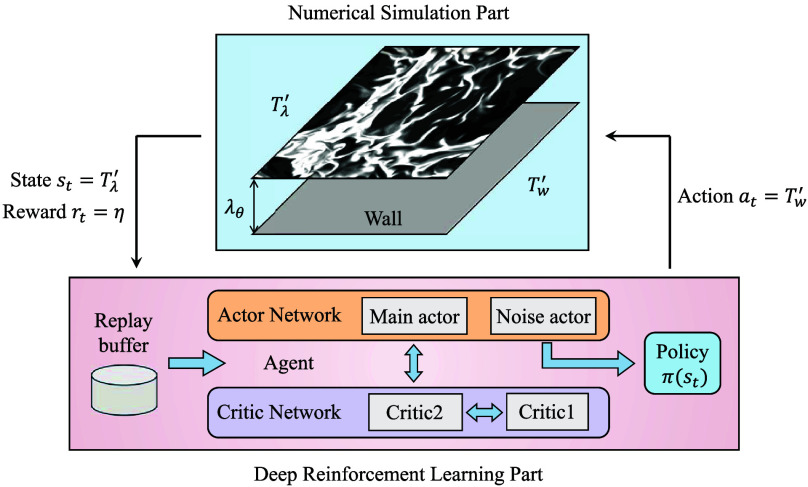
The flow chart of DRL-based control in turbulent convection. The numerical simulation environment provides state information and reward to the DRL agent, which optimizes its policy through actor–critic network and outputs the next action to control the flow field.

To optimize turbulent flow control, we employ DRL to govern the temperature fluctuations $T_{w}^{\prime }$, which constitute the control actions $a_{t}$. The control system consists of two core components: the numerical simulation and the DRL agent, as illustrated in Fig. 1. The simulation serves as the environment, providing both the instantaneous flow state $s_{t}$ and the reward $r_{t}$ that quantifies system performance. The DRL agent processes these inputs, iteratively updates its decision-making policy $\pi (s_{t})$, and generates actuation commands $a_{t}$ to manipulate the flow field. This closed-loop architecture enables adaptive flow control, with the agent continuously refining its policy through real-time interaction with the flow system. Within this framework, the wall temperature fluctuations $T_{w}^{\prime }$ are designated as control actions $a_{t}$, with amplitude constrained to the range $\left[-\Delta T,\Delta T\right]$. Meanwhile, temperature fluctuations $T_{\lambda }^{\prime }$ at the estimated thermal boundary layer height $\lambda_{\theta }=1/\left(2Nu_{0}\right)$ near the lower wall are selected as the states $s_{t}$, where $Nu_{0}$ denotes the baseline Nu in uncontrolled flow. The reward function $r_{t}$ is formulated as η, the relative enhancement of Nu compared to the baseline value, calculated from the mean heat flux on the lower wall. Through policy gradient optimization, the agent progressively enhances its control strategy to maximize the cumulative reward, thereby driving systematic performance improvement. Further details of the methodology are provided in *Materials and Methods*.

The heat transfer enhancement achieved through DRL-based control strategies is quantitatively characterized by the elevated Nusselt number $Nu_{d}$ and enhancement ratio $\eta_{d}$ shown in Table 1, while all results have been statistically convergent as described in *SI Appendix*. These strategies demonstrate sustained effectiveness at Ra up to $5\times 10^{8}$, with all cases exhibiting $\eta_{d}$ exceeding 30%, peaking at 38.5% when $Ra=5\times 10^{8}$. This performance significantly surpasses the maximum 20% to 25% enhancement ratio attainable through predefined sinusoidal temperature fluctuation controls (14).

**Table 1.** Computational parameters and heat transfer enhancement

Cases	*Ra*	$N_{x}\times N_{y}\times N_{z}$	$Nu_{0}$	$\lambda_{\theta }$	$Nu_{d}$	$\eta_{d}$(%)	$Nu_{s}$	$\eta_{s}$(%)
1E7	$1\times 10^{7}$	192×256×256	17.2	0.0291	23.4	35.8	23.2	35.1
1E8	$1\times 10^{8}$	288×384×384	32.5	0.0154	42.4	30.2	44.8	37.8
5E8	$5\times 10^{8}$	432×480×480	51.9	0.0096	71.9	38.5	71.1	36.8
1E9	$1\times 10^{9}$	512×576×576	64.0	0.0078	–	–	89.6	40.0

We display key simulation parameters for each case: Rayleigh number Ra, three-dimensional grid resolution $N_{x}\times N_{y}\times N_{z}$, baseline Nusselt number $Nu_{0}$ without control, and the estimated thermal boundary layer height $\lambda_{\theta }$. All cases maintain identical configuration parameters with Prandtl number Pr=1.0 and aspect ratio Γ=1. $Nu_{d}$ and $\eta_{d}$ denote the Nusselt number and heat transfer enhancement ratio achieved through DRL-based control strategies, respectively, while $Nu_{s}$ and $\eta_{s}$ represent their counterparts derived from the simplified bang-bang control strategy informed by DRL insights.

### Mechanism of Heat Transfer Enhancement.

**Fig. 2.**
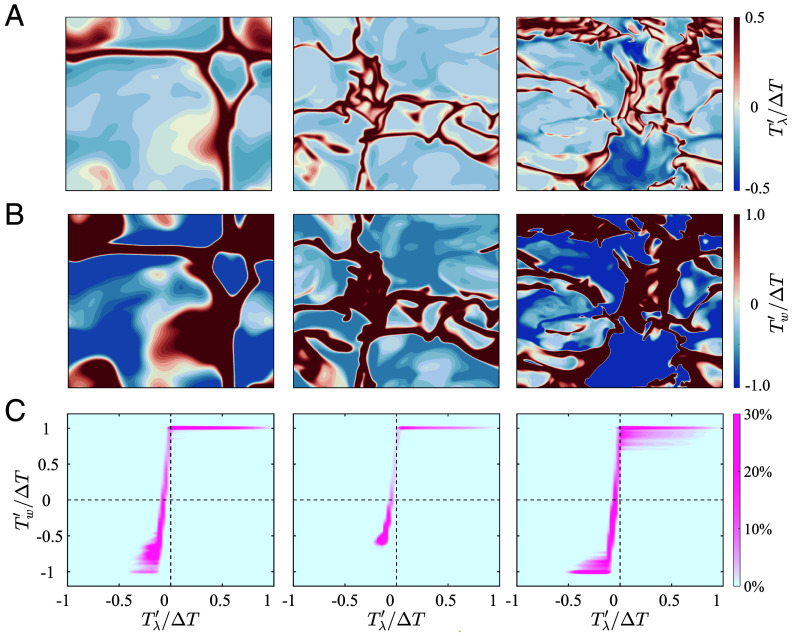
Relationship between input state and output action in DRL-based control strategy. From *Left* to *Right*: cases 1E7, 1E8, and 5E8. Instantaneous snapshots of temperature fluctuations in the y−z plane are shown at two locations: (*A*) the thermal boundary layer height $x=\lambda_{\theta }$, serving as the input state $T_{\lambda }^{\prime }/\Delta T$, and (*B*) the wall, representing the output action $T_{w}^{\prime }/\Delta T$. (*C*) The joint probability density function illustrates the statistical relationship between the input state $T_{\lambda }^{\prime }/\Delta T$ and the output action $T_{w}^{\prime }/\Delta T$, with contour plots showing the ratio relative to the maximum probability density.

Further investigations are warranted to elucidate the impact of DRL-based control on heat transfer and their underlying flow modification mechanisms. First, systematic analysis of the correlation between DRL-generated actuation signals and their corresponding input states is crucial, with their spatial distributions captured in Fig. 2 *A* and *B*. These instantaneous fields correspond to the flow state at 1,000 free-fall time units ($t_{0}=\sqrt{H/(\alpha g\Delta T)}$) after DRL-based control implementation, when the turbulent flow has been fully developed. The output wall temperature fluctuations $T_{w}^{\prime }$ generally exhibit the same sign as the input signal $T_{\lambda }^{\prime }$ at the corresponding horizontal locations. This phase alignment is accompanied by significant amplitude amplification, with distinct localized hot phase regions reaching the prescribed temperature actuation limit, ΔT. Notably, these characteristic hot phase regions persist while thermal structures become increasingly fragmented with elevated Ra, and the boundaries between hot and cold phases remain clear. The area of the cold phase near the lower wall substantially exceeds that of the hot phase in the input state fluctuations, replicating the spatial pattern characteristic of uncontrolled RB convection (37–39). The inherent thermal asymmetry propagates through the control process, ultimately generating comparable cold-hot phase area disparity in the resultant ($T_{w}^{\prime }$) distribution.

Fig. 2*C* presents the joint probability density function of normalized state–action pairs ($T_{\lambda }^{\prime }/\Delta T$, $T_{w}^{\prime }/\Delta T$), which statistically characterizes the input–output relationship in DRL-based thermal control strategies. Detailed information regarding the statistical samples is provided in Table 2 of *Materials and Methods*. The DRL-based control strategy consistently exhibits strong nonlinearity in the state–action relationship across all cases. In line with the observations in instantaneous fields, positive input states predominantly trigger positive outputs, which are highly concentrated around +ΔT. Conversely, as the input state temperature decreases and enters the negative part, the policy output action decreases rapidly, with characteristic cold-phase fluctuations centered around $T_{\text{cold}}^{\prime }$ above −ΔT. This asymmetric saturation behavior arises from the larger spatial coverage of the cold phase, necessitating reduced cold-phase fluctuation intensity to satisfy the zero-mean constraint of $T_{w}^{\prime }$. Interestingly, this control strategy resembles bang-bang control (40), which switches abruptly between extreme values. Such controllers have demonstrated remarkable effectiveness across diverse flow control applications, including wall-bounded turbulence (27) and airfoil roll control (41), where they outperform standard continuous control strategies in a wide class of fluid dynamics systems. This characteristic behavior is especially pronounced in our high-performance cases, where the cold-phase fluctuation $T_{\text{cold}}^{\prime }$ approaches the lower bound −ΔT. Furthermore, there exists a positive correlation between the magnitude of cold-phase fluctuations $|T_{\text{cold}}^{\prime }|$ and the heat transfer enhancement ratio η. In the highest-performing case 5E8 ($Ra=5\times 10^{8}$, η=38.5%), $T_{\text{cold}}^{\prime }$ approaches −ΔT, whereas in the lowest-performing case 1E8 ($Ra=1\times 10^{8}$, η=30.2%), the negative $T_{w}^{\prime }$ fluctuations are milder, with $T_{\text{cold}}^{\prime }$ around −0.6ΔT.

**Table 2.** Number of samples used for the statistics

Cases	$n_{y}$	$n_{z}$	$n_{t}$	$n_{\text{sum}}$	$t_{\text{sum}}/t_{0}$
1E7	256	256	150	$9.8\times 10^{6}$	1,500
1E8	384	384	150	$2.2\times 10^{7}$	1,500
5E8	480	480	100	$2.3\times 10^{7}$	1,000

We display the number of samples for each case: $n_{y}$, $n_{z}$, and $n_{t}$ denote sample numbers in the y, z, and t directions, and $n_{\text{sum}}=n_{y}n_{z}n_{t}$ is the total number of samples. $t_{\text{sum}}$ is the sampling time period.

The theoretical framework developed in previous studies on spatially modulated turbulent RB convection (14) provides a potential explanation for the heat transfer enhancement mechanisms observed in our DRL-based control strategy. These investigations identify two characteristic length scales governing convective heat transfer under temperature modulation (14): the penetration depth, which quantifies the vertical reach of control actions into the fluid domain, and the inversion depth, describing the wall-normal extent of the stable temperature inversion layer above cold phase regions. Heat transfer enhancement occurs through distinct mechanisms depending on the relative magnitudes of these length scales compared to the uncontrolled thermal boundary layer thickness $\lambda_{\theta }$. Penetration depths exceeding $\lambda_{\theta }$ enable hot-phase influences to penetrate beyond the boundary layer edge, inducing massive hot plume ejections. Furthermore, inversion depths surpassing $\lambda_{\theta }$ establish a fully modulated boundary layer regime where both hot and cold phases cooperatively reshape and destabilize the characteristics of the thermal boundary layer, ultimately amplifying the heat transfer.

**Fig. 3.**
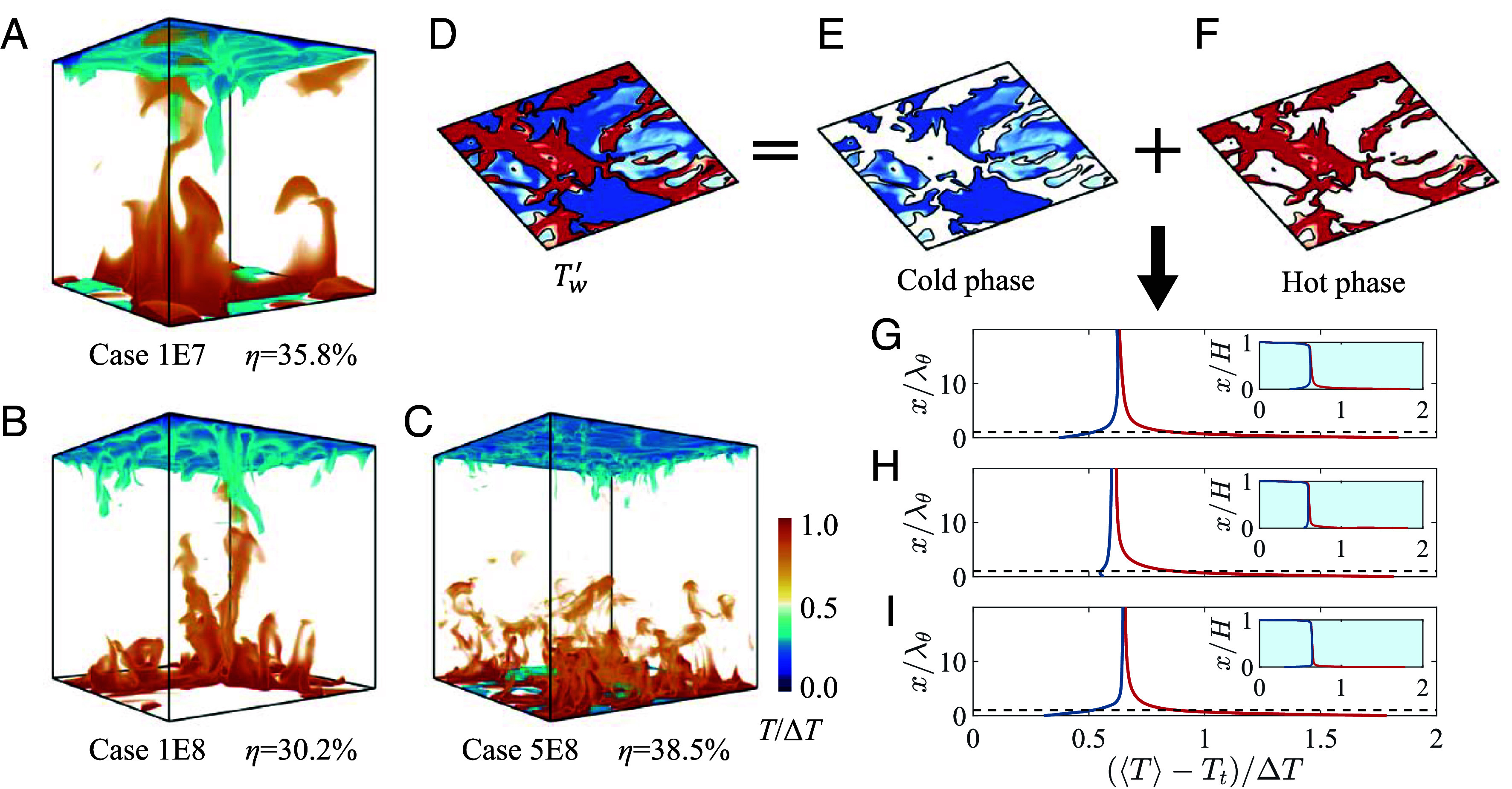
Effect of DRL-based control on turbulent convection flows. Instantaneous distributions of temperature iso-surfaces are shown for (*A*) cases 1E7, (*B*) 1E8, and (*C*) 5E8, where the high-temperature zone penetrates into the central flow region. (*D*) Wall temperature fluctuations $T_{w}^{\prime }$ are categorized into (*E*) cold phase and (*F*) hot phase based on their negative and positive values. The wall-normal distributions of the mean temperature $\langle T\rangle$ profiles, conditionally averaged based on the hot and cold phases, are presented for (*G*) cases 1E7, (*H*) 1E8, and (*I*) 5E8. The y-axis represents the wall-normal height x, with the main plot scaled by $\lambda_{\theta }$ and the *Inset* scaled by the cell height H. $T_{t}$ in the x-axis denotes the upper wall temperature used for nondimensionalization. Blue and red lines represent the temperature profiles over the cold and hot phases, respectively, with their differences reflecting the height of influence exerted by the DRL-based control. The black dashed lines in the main plot indicate the thermal boundary layer height $x=\lambda_{\theta }$.

The instantaneous temperature iso-surface distributions following DRL-based control (Fig. 3*A*–*C*) demonstrate substantial hot plume ejections across all cases, as evidenced by pronounced orange-yellow regions. These hot plumes exhibit deep penetration into the central flow region, with penetration depths under control significantly exceeding the thermal boundary layer thickness, also demonstrated in *SI Appendix*. Conditional averaging based on hot ($T_{w}^{\prime }>0$) and cold phases ($T_{w}^{\prime }<0$) of the temperature (Fig. 3*D*–*F*) provides a method to further quantify the relative magnitudes of the penetration depth and inversion depth compared to $\lambda_{\theta }$. The statistical samples used are consistent with those in Fig. 2, with details provided in Table 2 of *Materials and Methods*. The conditional averaged temperature profiles are presented in Fig. 3*G*–*I*. $\langle T\rangle$ represents the local average of temperature T in the horizontal direction and over time. In all cases, the local-averaged temperature profiles corresponding to hot and cold phases exhibit significant separation, extending beyond $10\lambda_{\theta }$. This indicates that the penetration depth far exceeds the thermal boundary layer thickness in all cases, suggesting that the DRL-based control can penetrate deeply into the central flow region. The inversion depth is defined as the height corresponding to the maximum temperature in the cold phase region. Although not clearly visible in Fig. 3, the inversion depth in all cases also exceeds $10\lambda_{\theta }$. Specifically, the inversion depths for cases 1E7, 1E8, and 5E8 are $12.7\lambda_{\theta }$, $21.8\lambda_{\theta }$, and $28.8\lambda_{\theta }$, respectively. These results demonstrate that the DRL-based control has triggered the fully modulated boundary layer regime, where the influences of both hot and cold phases extend beyond the thermal boundary layer and penetrate into the central flow region, leading to a substantial increase in heat transfer.

### Simplified Control Strategy from DRL Insights.

DRL offers an effective framework for optimizing control strategies to achieve superior heat transfer enhancement. While analytical formulation of flow control patterns derived through DRL typically proves challenging (19–22, 24–33, 42), the present study reveals a simple nonlinear state–action relationship, resembling a bang-bang controller. As demonstrated in Fig. 2*C*, this bang-bang control strategy exhibits a nonlinear step-like response behavior that outperforms conventional temperature modulation approaches. Specifically, the system generates an action of +ΔT when processing positive input states and approximately −ΔT for negative input states. This aligns with findings in prior fluid mechanics studies where bang-bang controllers emerge naturally in various flow control scenarios (27, 28, 41), suggesting they may represent fundamental optimal solutions for certain classes of turbulent systems. This observed correspondence between state and action also provides critical insights for developing simplified control architectures informed by DRL principles, while possibly maintaining comparable performance characteristics without any training process.

The derived control strategy inspired by DRL insights can be formulated as

**Fig. 4.**
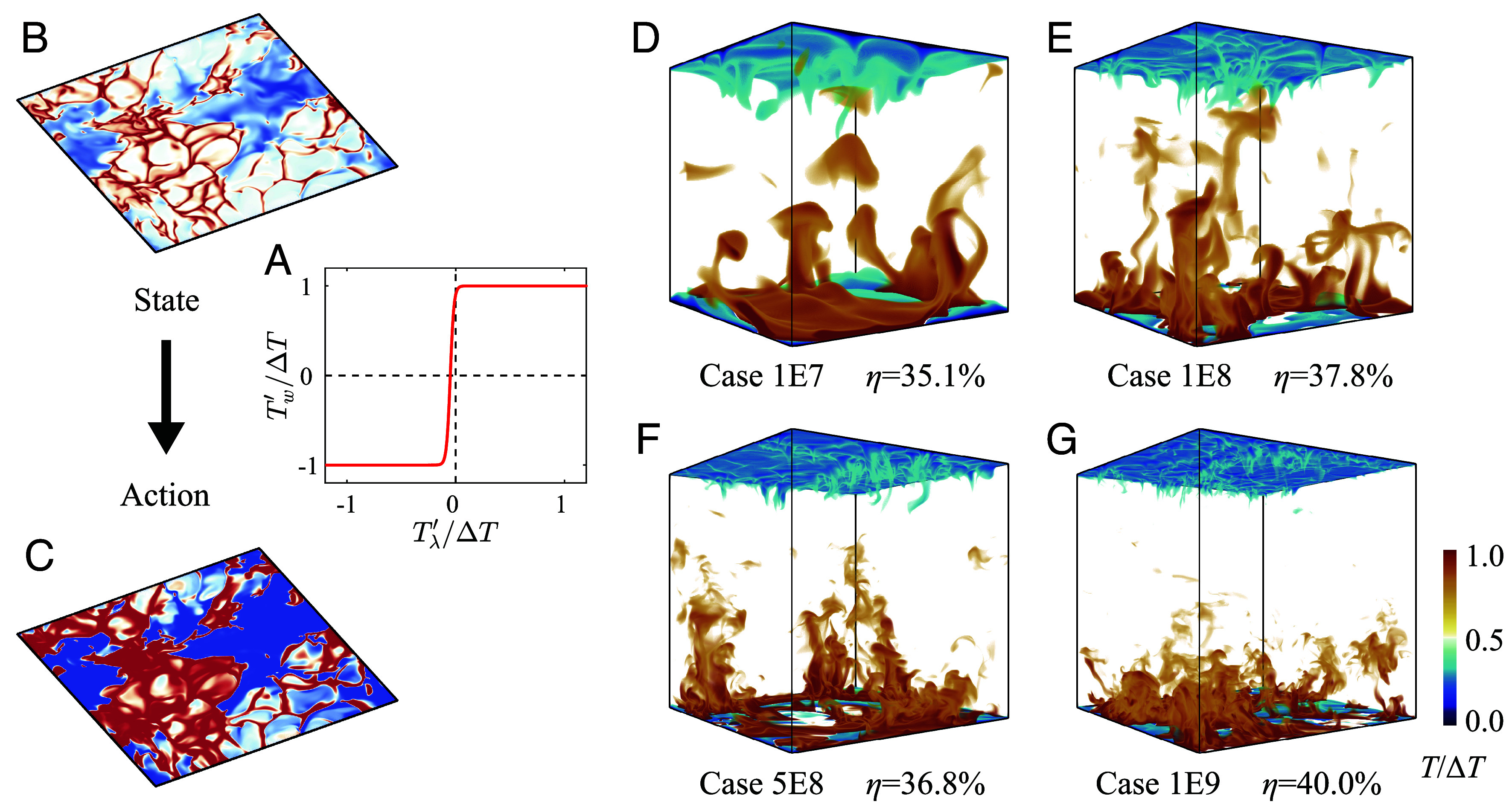
Simplified bang-bang control strategy and its outcomes. (*A*) The simplified hyperbolic tangent policy is adopted instead of the DRL agent, with (*B*) the input state and (*C*) the output action demonstrated for case 1E9 as an example. Instantaneous distributions of temperature iso-surfaces are shown for (*D*) cases 1E7, (*E*) 1E8, (*F*) 5E8, and (*G*) 1E9, showing the effectiveness of the control strategy up to $Ra=10^{9}$.

[1] $$T_{w}^{\prime }=\{\begin{array}{cc} \text{Δ}T & \left(T_{\lambda }^{\prime }>T_{0}\right)\\ -\text{Δ}T & \left(T_{\lambda }^{\prime }\leq T_{0}\right) \end{array}$$

constituting a standard bang-bang controller without hysteresis. Here, the threshold parameter $T_{0}$ ensures the sum of $T_{w}^{\prime }$ is zero by balancing the spatial contributions of heating and cooling phases. To address numerical singularities inherent in step functions, this simplified control strategy is revised using a hyperbolic tangent approximation (Fig. 4*A*–*C*):

[2] $$\begin{array}{c} T_{w}^{\prime }=\;\tanh\left[\beta (T_{\lambda }^{\prime }-T_{0})\right] \end{array}$$

The gain parameter β is chosen to emulate the DRL-based state–action relationship while ensuring numerical stability, with β=20 selected as a compromise between approximation fidelity and computational robustness. Posterior analysis confirms the robustness of Nu under control when $\beta \in \left[10,30\right]$. The threshold $T_{0}$ is determined through integral constraints to maintain zero mean wall temperature perturbation. This parameter primarily assumes negative values due to inherent asymmetries in the spatial distributions of heating and cooling phases.

The effectiveness of the distilled bang-bang control strategy is quantified in Table 1, where the enhanced Nusselt number $Nu_{s}$ and the corresponding improvement ratio $\eta_{s}$ are presented. Remarkably, the simplified model retains performance comparable to the DRL-derived control across all tested cases, with deviations in $\eta_{s}$ exceeding 2% only in case 1E8. In this particular case, the slightly lower enhancement observed in the DRL-based approach is linked to elevated $T_{\text{cold}}^{\prime }$ levels, as illustrated in Fig. 2*C*. The effectiveness of the distilled bang-bang control is further visualized in Fig. 4*D*–*G*, where the instantaneous temperature iso-surfaces reveal vigorous hot plume ejections penetrating deep into the central flow region-mirroring the mechanisms observed under DRL-based control. To assess the generalizability of the simplified strategy, we applied it to an unseen, higher Rayleigh number case at $Ra=10^{9}$. The strategy remained highly effective, achieving a heat transfer enhancement of 40.0%, demonstrating its robustness and scalability to more extreme turbulence regimes. This result highlights the potential of reinforcement learning in distilling interpretable and transferable control strategies for high-intensity turbulent convection.

### Performance with Downsampling.

Building on the proven effectiveness of our control framework, we now assess its performance under realistic implementation constraints. Practical limitations, including sensor placement density, measurement update frequency, actuator count, and signal processing delays, can significantly influence the effectiveness of control systems. To emulate these real-world constraints, we introduce the following engineered downsampling conditions: Sensors are uniformly distributed only along four edges of the horizontal cross-section at boundary layer height (16 sensors per edge), allowing feasible installations of sensors. Using these edge measurements, we then reconstruct the 2D temperature input with linear interpolation and update the edge temperature measurement periodically at intervals of $1.0t_{0}$ to reduce operational complexity (i.e., down-sampling temporally). Control actuation is implemented via a 16×16 wall-temperature actuator array (i.e., down-sampling spatially). In addition, we also incorporate a realistic signal-processing delay of $1.0t_{0}$. Further details and analyses of each constraint’s impact individually are available in *SI Appendix*. Remarkably, under these combined experimental constraints, in case 1E7, DRL-based control maintains heat transfer enhancements of 24.6%, while simplified bang-bang control achieves 22.3%. These results confirm the robustness of our proposed control methodology against practical sensing and actuation limitations. This resilience originates from the dominance of large-scale spatial patterns and slow-evolving temporal components in the control signals, as established in *SI Appendix*. Notably, even with this significant downsampling at much lower cost, both the DRL-based and simplified bang-bang control achieve heat transfer enhancement approaching the upper limit of the traditional sinusoidal heating control [around 23% (14)], which requires actuators operating at full DNS resolution without any spatial downsampling. This result further highlights the superior effectiveness and practical advantage of our control strategies over traditional methods. Taken together, the performance described above under realistic constraints strongly supports the potential applications of our method in real-world settings.

## Discussion and Conclusion

Our study demonstrates that DRL offers an innovative framework for optimizing heat transfer enhancement in high-Ra turbulent RB convection, achieving notable performance beyond conventional control strategies. By leveraging adaptive, nonlinear control policies, DRL-based thermal actuation generates sustained heat transfer improvements exceeding 30% at Rayleigh numbers up to $Ra=5\times 10^{8}$, significantly surpassing the 20 to 25% enhancement limits of predefined sinusoidal modulation methods.

A key finding lies in the distinct nonlinear state–action relationship governing the DRL-generated control strategy. Positive thermal fluctuations at the boundary layer trigger maximal heating +ΔT, while negative inputs yield milder cooling responses above −ΔT. This nonlinearity enables the system to amplify hot plume ejections while maintaining stable temperature inversion over cold-phase regions, critical for enhancing turbulent convection. The resulting fully modulated boundary layer regime, characterized by penetration and inversion depths exceeding $10\lambda_{\theta }$, confirms that DRL-driven actuation penetrates into the central flow region, leading to a significant increase in heat transfer.

The development of a simplified bang-bang control strategy, inspired by DRL insights, further underscores the robustness and scalability of this approach. By translating the learned nonlinear policy into a threshold-based hyperbolic tangent function, which constitutes a bang-bang controller without hysteresis, we achieved comparable heat transfer enhancements (e.g., 40.0% at $Ra=10^{9}$) without requiring iterative DRL training. This simplification retains the core mechanism of phase-aligned amplification while ensuring computational feasibility for further industrial applications. While sinusoidal controllers are widely studied for their mathematical convenience in linearized flow analysis, our work shows that bang-bang controllers-often distilled from DRL policies-are effective alternatives for turbulent flow control. These controllers represent a fundamental class of simple control laws deserving increased theoretical attention. This breakthrough also highlights the potential of machine learning to uncover control mechanisms that are finely attuned to the dynamics of turbulent flows, particularly in regimes where traditional approaches struggle to balance complexity and efficiency.

## Materials and Methods

### Deep Reinforcement Learning.

The DRL framework illustrated in Fig. 1 employs the twin-delayed deep deterministic policy gradient (TD3) algorithm (43), utilizing an open-source implementation tailored for turbulence control by Lee et al. (30). Both input states and output actions are represented as two-dimensional arrays with dimensions $N_{y}\times N_{z}$, while the instantaneous reward corresponds to the relative Nu enhancement. The TD3 model is an actor–critic network architecture designed to enhance learning stability and performance by addressing overestimation bias, employing delayed updates, and applying target smoothing techniques. During the training process, the TD3 algorithm aims to optimize the action value function $q_{\pi }\left(s_{t},a_{t}\right)$ through satisfaction of the Bellman equation:

[3] $$\begin{array}{c} q_{\pi }\left(s_{t},a_{t}\right)=E\left[r_{t}^{d}+\gamma^{n}q_{\pi }\left(s_{t+n},\pi_{\phi }\left(s_{t+n}\right)+\epsilon \right)\right], \end{array}$$

where $r_{t}^{d}=\sum_{j=1}^{n}\gamma^{j-1}r_{t+j}$ denotes the n-step discounted reward, γ the discount factor, $\pi_{\phi }\left(s_{t+n}\right)$ the delayed policy update, and ϵ the clipped random noise. Parameters are set to n=5 and γ=0.95 following the open-source code. The critic networks estimate the expected cumulative reward, with their parameters updated via the objective function:

[4] $$\begin{array}{c} J\left(\theta \right)=N^{-1}\sum {\left[r_{t}^{d}+\gamma^{n}q_{\pi }\left(s_{t+n},\pi_{\phi }\left(s_{t+n}\right)+\epsilon \right)-q\left(s_{t},a_{t}\right)\right]}^{2}, \end{array}$$

where N=64 represents the minibatch size, and θ is the critic network weights. The actor network seeks to determine an optimal policy, guided by the policy objective function J(ϕ), with ϕ denoting the weight parameters of either network. The objective function is ultimately optimized through:

[5] $$\begin{array}{c} \nabla_{\psi }J\left(\psi \right)=E\left[\nabla_{a}q_{\pi }\left(s_{t},a_{t}\right){|}_{a=\pi (s_{t})}\nabla_{\psi }\pi_{\psi }\left(s_{t}\right)\right], \end{array}$$

where ψ denotes the actor network weights.

The network architectures in the DRL framework are structured as follows (30): The actor network consists of three convolutional layers, which progressively reduce feature complexity by employing 64, 32, and 1 filters respectively, with each filter kernel sized at 3×3. In contrast, the critic network adopts a deeper architecture comprising six convolutional layers followed by three fully connected layers. Each convolutional layer contains 32 filters of size 3×3. To enhance feature abstraction, an average pooling layer is strategically inserted after every pair of convolutional layers. The subsequent fully connected layers maintain dimensional consistency with 32 neurons per layer, ultimately producing a scalar q-value output to assess the quality of the control policy. The ReLU activation function is applied uniformly across all layers in both networks.

The training process requires balancing temporal development of control strategy adjustments with computational efficiency. We establish each state step duration as five free-fall time units ($5t_{0}$) to permit sufficient flow response, while limiting episodes to $100t_{0}$ (20 sequential state steps) to constrain computational costs. This configuration implements policy updates every 5 steps, ensuring alignment between gradient updates and the n-step reward horizon. During the training phase of the control strategy, we formulate the normalized reward $\bar{r}$ as $\bar{r}=\sum_{j=1}^{n}\gamma^{j-1}r_{t+j}/\sum_{j=1}^{n}\gamma^{j-1}$. This metric serves to assess the algorithm’s learning performance. Oscillations in $\bar{r}$ around low-reward value primarily occur within the initial 10 training episodes. Subsequently, the normalized reward rapidly increases and reaches a high-reward plateau, with the metric stabilizing to demonstrate convergence of the DRL models, also documented in *SI Appendix*. Across all cases, the maximum $\bar{r}$ values appear within the high-reward plateau range of 10 to 25 episodes. As suggested by Lee et al. (30), extended training periods can result in issues such as catastrophic forgetting or overfitting, which could potentially lead to training failure. Thus, the final control strategy is chosen based on the episode achieving the peak $\bar{r}$ value.

Further implementation details of the DRL framework can be found in *SI Appendix* and Lee et al. (30). In addition, the code for the DRL-based control is shared via GitHub at https://github.com/zhouzisong1997/DRL_RB_control (44).

### Direct Numerical Simulations.

We consider RB convection established between two parallel plates, with the fluid being heated from below and cooled from above. The governing equations are the three-dimensional incompressible Navier–Stokes equations within the Boussinesq approximation, written as

[6] $$\begin{array}{c} \nabla \cdot u=0, \end{array}$$

[7] $$\begin{array}{c} \frac{\partial u}{\partial t}+u\cdot \nabla u=-\nabla P+\nu \nabla^{2}u+\alpha gT\hat{x}, \end{array}$$

[8] $$\begin{array}{c} \frac{\partial T}{\partial t}+u\cdot \nabla T=\kappa \nabla^{2}T, \end{array}$$

where u is the velocity vector, P is the kinematic pressure, and $\hat{x}$ is the wall-normal unit vector. The flow is assumed to be periodic in the horizontal directions. No-slip and no-penetration boundary conditions are imposed on the two walls, where the velocity is set to u=0. The top wall is maintained at a constant temperature $T_{t}$, while the bottom wall is subjected to temperature modulation with a temperature of $T_{b}+T_{w}^{\prime }\left(y,z,t\right)$.

Direct numerical simulations were performed using the AFiD code (45–47). The numerical method employs an energy-conserving, second-order finite difference scheme for spatial discretization, with velocities on a staggered grid. Time marching is achieved through a third-order Runge–Kutta scheme, complemented by a Crank-Nicolson method for implicit terms. The computational grid features uniform spacing in the horizontal directions, while employing clipped Chebyshev-type clustering in the wall-normal direction to resolve boundary layer dynamics near the plates. This code has been extensively validated in previous RB convection studies (45–50).

In this study, all the cases discussed have reached a fully developed turbulent state within $500t_{0}$ following control implementation. Consequently, all statistical averaging commences from $500t_{0}$ after the control is applied. To account for the fluctuations in Nu measurements, a temporal averaging window of $1000t_{0}$ is applied to obtain each Nu in Table 1, ensuring statistical stationarity with a sample size of $10^{5}$ for each case. The sample numbers for the state–action map (Fig. 2*C*) and temperature conditional averaging (Fig. 3*G*–*I*) are summarized in Table 2.

## Supplementary Material

Appendix 01 (PDF)

Movie S1. **Flow field evolution (*Ra* = 10^7^) with and without control**. Evolution of temperature isosurfaces for the flow in case 1E7. The left part shows the non-actuated case, while the right part represents the case after DRL-based control. Both sides start from the same initial state. Compared to the non-actuated case, the DRL-based control induces significant hot plume ejections originating from the lower wall and penetrating deep into the central flow region.

Movie S2. **Flow field evolution (*Ra* = 10^8^) with and without control**. Evolution of temperature isosurfaces for the flow in case 1E8. Descriptions of the flow dynamics and control effects are the same as Movie S1.

Movie S3. **Flow field evolution (*Ra* = 5 × 10^8^) with and without control**. Evolution of temperature isosurfaces for the flow in case 5E8. Descriptions of the flow dynamics and control effects are the same as Movie S1.

## Data Availability

Code for the DRL-based control has been deposited in GitHub (https://github.com/zhouzisong1997/DRL_RB_control) (44). All other data are included in the manuscript and/or supporting information.
