# Supplementary material for: Deep reinforcement learning control unlocks enhanced heat transfer in turbulent convection
Source: Proc Natl Acad Sci U S A. 2025 Sep 9;122(37):e2506351122. doi: 10.1073/pnas.2506351122 (PMC12452834; doi:10.1073/pnas.2506351122)
Supplement: Supplementary file 1 — Appendix 01 (PDF) [file pnas.2506351122.sapp.pdf]

PNAS

Supporting Information for

Deep reinforcement learning control unlocks enhanced heat transfer in turbulent convection

Zisong Zhou and Xiaojue Zhu

Xiaojue Zhu.
E-mail: zhux@mps.mpg.de

This PDF file includes:

Supporting text
Figs. S1 to S5
Tables S1 to S6
Legends for Movies S1 to S3
SI References

Other supporting materials for this manuscript include the following:

Movies S1 to S3

Supporting Information Text

Further details for deep reinforcement learning

This section supplements the metaparameters and implementation details of the deep reinforcement learning (DRL) process. The metaparameters used in the deep reinforcement learning process are listed in Table S1, primarily following the optimized results from Lee et al. (1). Additionally, the code for the DRL-based control is shared via GitHub at https://github.com/zhouzisong1997/DRL_RB_control.

In Equation 3 (action value function) of the main text, clipped random noise ϵ is introduced into target policy smoothing regularization to mitigate overfitting, defined as $\epsilon \sim \mathrm{Clip}(\mathcal{N}(0,\sigma_q), -c, c)$. Here, $\mathcal{N}(0,\sigma_q)$ represents random numbers sampled from a zero-mean Gaussian distribution with a standard deviation of σ_q, and c denotes the clipping range. A discount factor of $\gamma = 0.95$ was employed during training, consistent with Lee et al.'s methodology (1), to accelerate convergence by prioritizing immediate rewards. Given our 5-step reward configuration, the theoretically optimal range for γ is determined to be $0.95 \sim 0.99$. Posterior analysis confirms that for turbulent convection scenarios, γ values within this range show robust results, which yield convergent solutions with comparable rewards.

The DRL framework employs the twin-delayed deep deterministic policy gradient (TD3) algorithm, which necessitates an exploration noise strategy to circumvent local optimum convergence. Consistent with Lee et al. (1), we employ Ornstein-Uhlenbeck (OU) process-based noise for exploration. This parameter-space noise exhibits temporal correlation and demonstrates superior performance in high-dimensional action spaces. Lee et al. (1) further established that OU-process noise effectively reduces non-physical reward fluctuations and enhances training convergence compared to random-walk alternatives. The specific computation proceeds as follows. First, the noise intensity requires adaptive scaling as the environment changes. Consequently, the relationship between the standard deviation for exploration noise at the next timestep σ_{t+1} and the current standard deviation σ_t satisfies:

$$\sigma_{t+1} = \begin{cases} \alpha\sigma_t & (d < \lambda) \\ \sigma_t/\alpha & (d \geq \lambda) \end{cases} \qquad [1]$$

Here, the distance $d(\pi, \widetilde{\pi})$ between the perturbed policy $\widetilde{\pi}$ and the main policy π is defined as:

$$d(\pi, \widetilde{\pi}) = \sqrt{\frac{1}{N}\sum_{i=1}^{N} \mathbb{E}\left[\pi(s_t)_i - \widetilde{\pi}(s_t)_i\right]^2}. \qquad [2]$$

The metaparameters α and λ denote the adaptive scaling factor and the threshold value of the disturbed distance, respectively, with their assigned values provided in Table S1. Then, the temporally correlated OU-process noise is calculated through:

$$dx_t = \xi(\mu - x_t)dt + \sigma_t \mathcal{N}(0, \sqrt{dt}), \qquad [3]$$

where ξ and μ represent the mean reversion constant and the mean value of the OU-process noise, respectively, shown in Table S1. Since the noise is added to the main actor's weights by the noise actor, updates to the noise actor are required whenever the main actor undergoes an update. Thus, the noise actor's update rate is configured to 5 , aligning it with the target update frequency.

The TD3 algorithm implements an actor-critic framework. Figure S1 depicts the specific actor-critic architecture employed in this study, adopting the configuration established by Lee et al. (1). The input state comprises an array of dimensions $[N_y, N_z]$, corresponding to sensors distributed across all grid points of the horizontal cross-section at boundary layer height. The output action maintains identical dimensions to the state, with actuators uniformly arranged at every grid point on the wall surface. In the actor-critic network, all convolutional layers utilize filter kernels sized 3×3. The actor network is structured with three convolutional layers employing 64, 32, and 1 filters respectively to progressively refine feature extraction. This architecture transforms input temperature fluctuations into wall temperature perturbations while preserving spatial dimensions. It should be noted that we implemented no further spatial or temporal smoothing on the output wall temperature perturbations. Lee et al. (1) pointed out that the three-convolution-layer structure proved sufficient for optimal performance, as adding more hidden layers or enlarging the input domain yielded no measurable gains. This is also consistent with our observations in turbulent convection systems. In contrast, the critic network incorporates a deeper topology: six convolutional layers and three fully connected layers. Average pooling is performed after every two convolutional layers. Following convolution, feature maps undergo flattening before passing through two fully connected layers, ultimately generating a scalar Q-value.

Building upon our actor-critic network architecture, we contextualize our approach within Belus et al.'s DRL framework classification (2): Method 1 (M1) globally concatenates and flattens all input regions before policy processing; Method 2 (M2) processes spatially distributed inputs through translationally invariant convolutional networks with shared weights; Method 3 (M3) decomposes the domain into discrete multi-agent environments with individualized rewards, also known as multi-agent reinforcement learning (MARL) (2, 3). Notably, our implementation chooses M2, motivated by three considerations. First, the statistical homogeneity observed in fully developed turbulent flow suggests that optimal control policies may exhibit translational invariance, which is also supported by the horizontal periodic boundary conditions implemented in our system. Second, convolutional architectures appear advantageous for preserving coherent turbulent structures through their geometric feature extraction capabilities, whereas localized point-based actuation could potentially struggle to maintain structural integrity due to its limited receptive field. Third, in the complex non-linear turbulent systems, locally optimized actions might not

necessarily translate to global performance gains. Training results (see Table 1 of the main text) confirm our M2 implementation achieves significant heat transfer enhancement, even the localization property of the reward is lost. Furthermore, compared to MARL, the convolutional neural network (CNN)-based approach reduces the parameter space dimensionality through weight-sharing mechanisms, which enhances computational efficiency during both training and deployment phases.

The current code implementation employs the multi-environment approach shown in Table S1, a widely applied treatment in DRL (4). As illustrated in Figure 1 of the main text, the codebase comprises two core components: the DRL module and the DNS module. The DRL module, developed in Python, builds upon the open-source framework provided by Lee et al. (1). It utilizes TensorFlow as its underlying DRL framework and operates within the specific Python environment defined by Palanisamy (5). The DNS module, implemented in Fortran, is based on the widely adopted open-source code AFiD (6–8). Training is executed exclusively on CPU resources. Due to its lower computational demands, the DRL part runs as a single-core serial process. Conversely, the DNS part operates as an MPI-parallelized program. During execution, the DRL module acts as the main program, responsible for initiating, terminating, and resetting the DNS simulations. Data exchange between the modules is facilitated through external storage. The DRL module writes actions to an HDF5 file on the CPU node's local SSD, which the DNS module subsequently reads. Following simulation, the DNS module writes the resulting state (to an HDF5 file) and the reward value (to a txt file) back to the local SSD, where they are read by the DRL module for the next process.

All training was exclusively CPU-based utilizing Intel Cascade Lake processors. For the 1E7, 1E8, and 5E8 cases, we conducted 10 independent random-seed experiments per case group, selecting optimal-performing seeds for final analysis. Computational resources allocated per seed were 288, 672, and 1056 CPU cores respectively, with corresponding wall-clock times of 0.11s, 0.25s, and 0.26s per time step advancement. Notably, the DRL component required < 0.01s per step under single-core serial execution, consistently below 10% of total step time across all cases. This indicates DNS computation dominated processing overhead, consistent with the prior research (4). Given that the AFiD-based DNS solver demonstrates strong parallel scaling efficiency with increasing problem size (6–8), our framework retains capacity for further scaling to larger-scale DRL-controlled turbulent systems. For the 1E7, 1E8, and 5E8 cases, aggregate wall-clock durations for the ten seeds were 306h, 695h, and 1445h, consuming total hardware-time resources of 88k, 467k, and 1.53M core-hours respectively.

Confirming the training status of the DRL models is essential. The dual evolution of the normalized reward $\bar{r}$ and critic loss, illustrated in Figure S2, provides quantitative metrics for assessing training convergence. Defined as $\bar{r} = \sum_{j=1}^{n} \gamma^{j-1} r_{t+j} / \sum_{j=1}^{n} \gamma^{j-1}$, the normalized reward serves as an indicator of learning performance throughout control policy training, where $r = \eta$ represents the relative enhancement in Nu compared to the baseline. Figure S2 A depicts the evolution of $\bar{r}$ across episodes for various cases. Prior to Episode 10, $\bar{r}$ manifested sustained oscillations near low values. Subsequently, during Episodes 10–15, it increased rapidly before reaching a plateau near 0.4, consistently maintaining high values thereafter. Posterior verification confirmed that control policies extracted post-plateau yielded robust heat transfer enhancement, indicative of training convergence. Complementarily, the critic loss quantifies the error in the critic network's evaluation of state-action value (Q-value), calculated as the mean squared difference between predicted Q-values and the temporal difference target derived from the Bellman equation. As shown in Figure S2 B, critic loss values begin to be recorded only after the replay buffer is populated (denoted as State 0), subsequently exhibiting a sharp decline during training and achieving convergence across all cases. This stabilization of critic loss directly reflects the convergence of Q-values towards accurate and consistent predictions of future flow control rewards, further corroborating the overall training process convergence.

Actuated vs. non-actuated cases

This section supplements detailed results and statistics for all cases in the main text, with a primary focus on the comparison between actuated and non-actuated cases.

First, we supplement the temporal evolution of Nu for all cases in the main text, shown in Figure S3. Time $t = 0$ marks the onset of control in actuated cases, with the Nu behavior prior to this instant being identical to non-actuated cases. Since control is applied directly to the lower wall, Nu at the lower wall (red lines) exhibits immediate changes upon actuation, followed by a gradual increase at the upper wall (blue lines) during the transient phase. This transient period lasts approximately $50t_0$ in case 1E7, $100t_0$ in case 1E8, $200t_0$ in case 5E8, and $300t_0$ in case 1E9, with duration increasing at higher Ra. Subsequently, the flow transitions to an established regime where turbulence becomes fully developed. After $500t_0$ of control application, all cases achieve statistically convergent Nu values at both walls. All statistical results are calculated based on this established regime, with Nu values obtained from statistical averaging between $500t_0$ and $1500t_0$ after control initiation.

Compared to non-actuated cases, the DRL-based control strategy exerts a significant influence on the central flow region, manifesting as strong hot plume ejections, as shown in Figure 3 of the main text. To quantify this phenomenon, we statistically analyzed the wall-normal distance distribution of temperature isosurfaces from the lower wall. Focusing on hot plume ejections, we selected the $T = 0.7\Delta T$ isosurface for analysis, where the upper plate temperature serves as the $T = 0$ reference baseline. The probability density function (p.d.f.) of its height distributions above the lower plate is presented in Figure S4. A posteriori verification confirmed qualitatively robust results for isosurfaces with $0.6\Delta T < T < 0.8\Delta T$. Relative to non-actuated cases, hot plume isosurfaces under DRL control exhibit markedly stronger clustering within the central flow region, with probability densities differing by over an order of magnitude ($> 10\times$). This quantitative result demonstrates that the DRL-based control elevates the height of the hot plume ejections in the flow field, consistent with observations from instantaneous flow visualizations.

Supplementary Movies S1–S3 compare temperature isosurface evolution in flows at $Ra = 10^7$, 10^8, and 5×10^8, contrasting non-actuated (left) and DRL-controlled cases (right) from identical initial states. For clarity in visualization, asymmetric

isosurface values were selected for hot versus cold regions, to prevent excessive volumetric dominance of hot structures after control. Upon actuation, significant hot plume ejections emerge near the wall across all cases; these structures progressively penetrate into higher flow regions over time, ultimately reaching the central domain.

Feasibility analysis for flow control

This study demonstrates the potential of DRL combined with simplified models in optimizing turbulent Rayleigh-Bénard (RB) convection heat transfer, achieving a remarkable enhancement efficiency of 38.5%. While these results highlight the theoretical viability of the approach, practical implementation challenges emerge regarding three critical aspects: the feasibility of real-time near-wall temperature measurements, the technical complexity of high-resolution actuator arrays, and the impact of control signal time delays. To systematically assess the robustness of our control strategy under operational constraints, we conducted targeted numerical experiments incorporating realistic limitations. The experimental framework introduces practical constraints by modifying and downsampling both input parameters (temperature fluctuations at the boundary layer height, T'_λ) and output parameters (wall temperature fluctuations, T'_w). Through quantitative analysis of the heat transfer enhancement coefficient η under these conditions, we provide a comprehensive evaluation of the methodology's applicability in real-world thermal management systems.

Input measurement. First, real-time high-resolution temperature measurements near the boundary layer height face significant technical barriers due to measurement accessibility constraints. Whereas detailed central-zone temperature measurements are challenging, temperature fluctuations only along the four edges of the cross-sectional boundary layer demonstrate greater measurability in operational environments (9). To align with practical sensing capabilities, we acquire temperature data through an array of uniformly distributed sensors positioned along each edge, featuring n_m measurement points per side. The two-dimensional temperature fluctuation field T'_λ is reconstructed through a dual-stage linear interpolation approach. First, the one-dimensional linear interpolation along each edge establish the boundary values. Then, interior points are estimated via a bidirectional weighted averaging scheme that integrates contributions from all four reconstructed boundaries.

The influence of constrained temperature measurement on heat transfer enhancement η is examined in Table S2, with the simplified hyperbolic tangent control strategy serving as a representative test case. The full resolution scenarios correspond to configurations where sensors are deployed at every grid point along all four edges. Here, sensor numbers exceeding $n_m = 16$ demonstrate a trivial impact on η values. Critically, even reconstructing the T'_λ field through a relatively crude linear interpolation, all tested configurations sustain heat transfer enhancement exceeding 20%. This observation confirms the robustness of the proposed control strategy, as it maintains sufficient effectiveness under both spatially limited sensor distributions and reduced measurement resolutions. Furthermore, this suggests potential for further heat transfer enhancement through advanced reconstruction techniques.

On the other hand, real-time temperature measurements significantly increase control costs, whereas temporally periodic data sampling can effectively reduce operational complexity. Therefore, we implement a control framework where temperature measurements occur at discrete intervals $\triangle t_m$ (temporal down-sampling), with wall temperature adjustments executed using the most recent measurements. Table S3 quantifies the hyperbolic tangent control strategy's performance across different time intervals, normalized against the free-fall time t_0. To contextualize t_0 magnitudes in practical systems, we assume the working fluid is water at 30°C between vertical plates spaced 20 cm apart. This configuration yields free-fall times of $t_0 = 36.7s$ at $Ra = 1 \times 10^7$ and $t_0 = 11.6s$ at $Ra = 1 \times 10^8$. Notably, despite decreasing heat transfer enhancement with increasing measurement intervals in table S3, the thermal enhancement η remains above 20% even at measurement intervals up to $5t_0$. This confirms that the control strategy could preserve control effectiveness while reducing costs through temporal down-sampling.

Output actuator. The implementation challenges encompass not only measurement constraints but also actuator configuration complexities governing wall temperature regulation. The high hardware costs of DNS-grid-matching actuator arrays pose a major barrier. To address this limitation, we tested the control strategy under actuator configurations with reduced spatial resolutions (i.e., spatial down-sampling). The wall surface is subdivided into a grid of $n_c \times n_c$ uniformly distributed control elements, each governing a square subdomain with edge length L/n_c, where L represents the computational domain's horizontal length. Within this framework, each actuator outputs the spatially averaged temperature fluctuation T'_w within its own subdomain. Table S4 shows the heat transfer enhancement dependence on actuator density, with full-resolution grid-matched cases providing baseline performance metrics. As the number of actuators decreases, heat transfer enhancement progressively diminishes. However, the control strategy maintains enhancement levels above 20% even with actuator counts reduced to an 8×8 configuration, highlighting its notable resilience. This analysis reveals that the methodology tolerates spatial down-sampling of actuator arrays while retaining functional efficacy, lowering implementation barriers for practical thermal management systems.

The temporal delay between actuator responses and measurement signals presents another critical implementation consideration in practical control systems. We assess the heat transfer enhancement under different time delays $\triangle t_c$ between sensor input and actuator output, as quantified in Table S5. Although heat transfer enhancement diminishes with increasing time delay, the control strategy still maintains enhancement above 20% even at $\triangle t_c = 5t_0$, demonstrating sustained control effectiveness.

Integrated real-world performance evaluation. The practical implementation of thermal control strategies requires simultaneous consideration of multiple operational constraints, necessitating a comprehensive evaluation of the proposed methodologies under

coupled real-world conditions. We implement representative experimental parameters incorporating concurrent limitations: Temperature monitoring utilizes $n_m = 16$ uniformly distributed sensors only along the four edges of the cross-sectional boundary layer, with measurement updates at intervals of $\triangle t_m = t_0$ with temporal down-sampling. The control system incorporates a latency of $\triangle t_c = t_0$ between sensing and actuation, while thermal regulation is achieved through a full-domain 16×16 actuator array with spatial down-sampling ($n_c = 16$). This configuration maintains experimental feasibility with significantly reduced implementation requirements. Under these simulated operational conditions, both the deep reinforcement learning model and simplified bang-bang strategy deliver significant heat transfer enhancement at $Ra = 10^7$, as quantified in Table S6. Specifically, the DRL-based control strategy yielded an enhancement of 24.6%, while the simplified bang-bang control attained 22.3%. Critically, even with substantial downsampling at significantly lower cost, both approaches attain enhancement approaching the performance limit (around 23% (10)) of traditional sinusoidal control, which demands actuators operating at full DNS resolution without spatial downsampling. These results demonstrate the experimental viability and broad generalization potential of our strategies under complex real-world constraints. They further highlight the superior practical advantage of our approach over traditional methods.

Mechanism behind. The persistent effectiveness of the control strategy under realistic operational constraints can be attributed to the inherent spatial and temporal characteristics of the wall temperature signals. Figure S5 A displays the premultiplied horizontal energy spectra kE_T of T'_w, where k denotes the horizontal wavenumber and $\lambda = 2\pi/k$ represents the corresponding wavelength. Analysis reveals that large-scale fluctuations at $\lambda > 0.1H$ dominate the energy spectra across two tested Ra values, significantly exceeding small-scale contributions. This spectral dominance explains the strategy's resilience to spatial coarsening, as practical limitations in sensor placement and actuator resolution primarily attenuate small-scale spatial information, while preserving the critical large-scale components governing the control mechanism. Temporal continuity is quantified through the correlation coefficient R, calculated between $T'_w(t)$ and $T'_w(t + \triangle t)$ across all statistical samples. Figure S5 B demonstrates a gradual decline in R with increasing $\triangle t$, yet maintains values above 0.5 even at $\triangle t = 10t_0$. This slow temporal evolution reflects the predominance of low-frequency signals in the control strategy, enabling robustness against non-real-time measurements and actuation delays. Collectively, these findings reveal that the control strategy mainly relies on large-scale spatial patterns and slowly evolving temporal signals. The preservation of these essential characteristics in practical implementations explains the sustained performance of our control strategies.

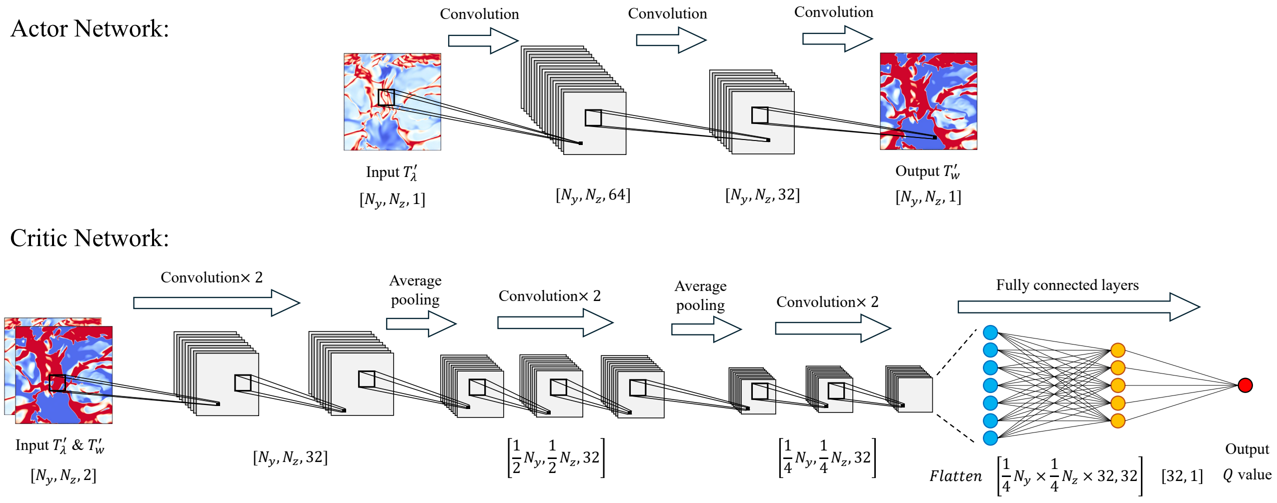

Fig. S1. The architecture of the actor-critic network.

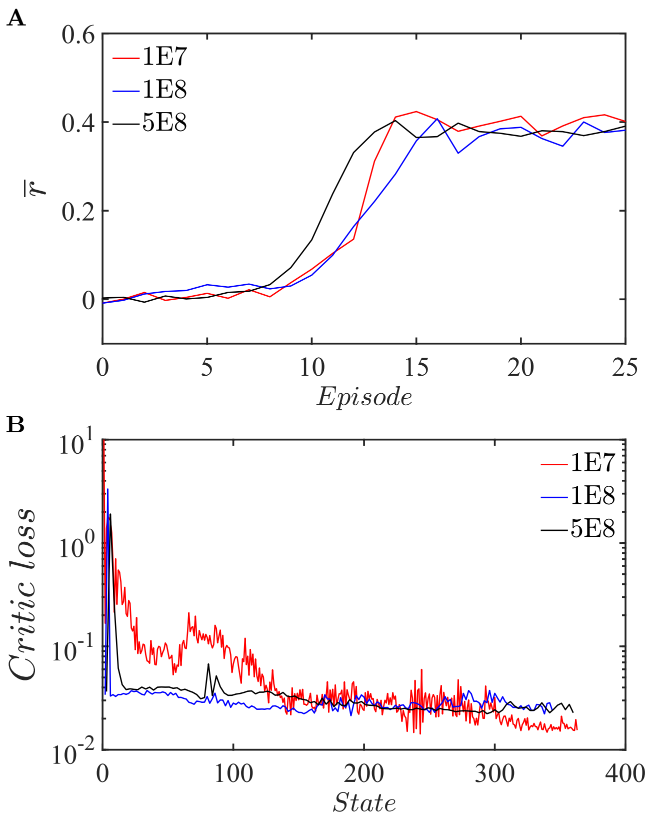

Fig. S2. Convergence diagnostics of the DRL agent. (A) Evolution of the normalized reward during the training process. (B) Evolution of the critic loss during the training process.

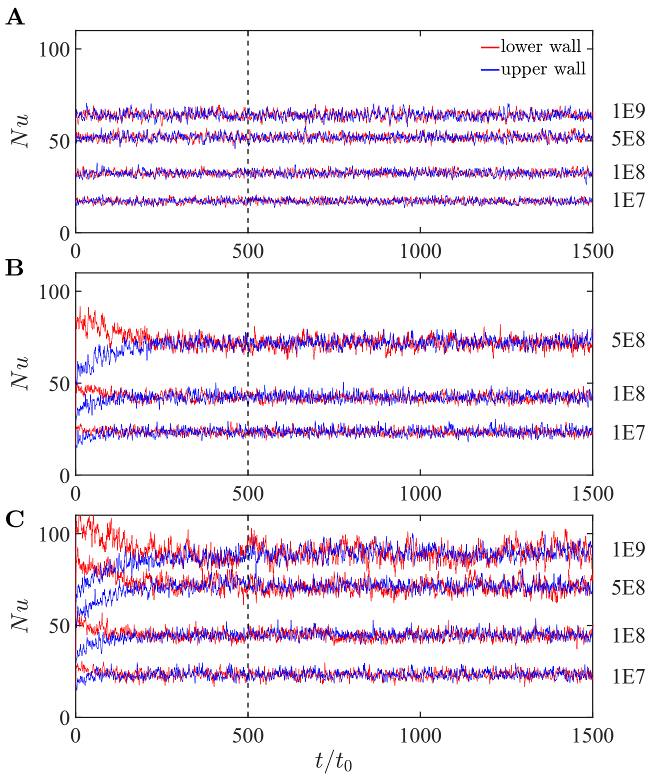

Fig. S3. Time evolution of Nu in all cases. (A) Non-actuated cases; (B) Cases with DRL-based control; (C) Cases with simplified hyperbolic tangent control. Red lines represent the lower-wall Nu; blue lines correspond to upper-wall Nu. Both become statistically identical after full flow development. Case names for each pair of curves are listed on the right side of the figure.

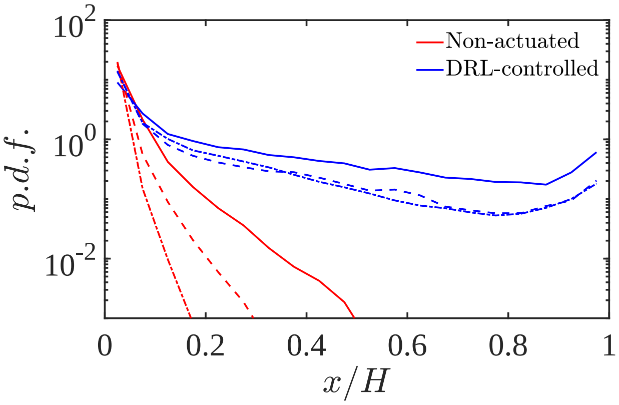

Fig. S4. Probability density function ($p.d.f.$) of the wall-normal distance distributions of the $T = 0.7\Delta T$ isosurface from the lower wall. Red and blue curves represent non-actuated cases and DRL-controlled cases, respectively; solid, dashed, and dash-dotted lines correspond to cases 1E7, 1E8, and 5E8.

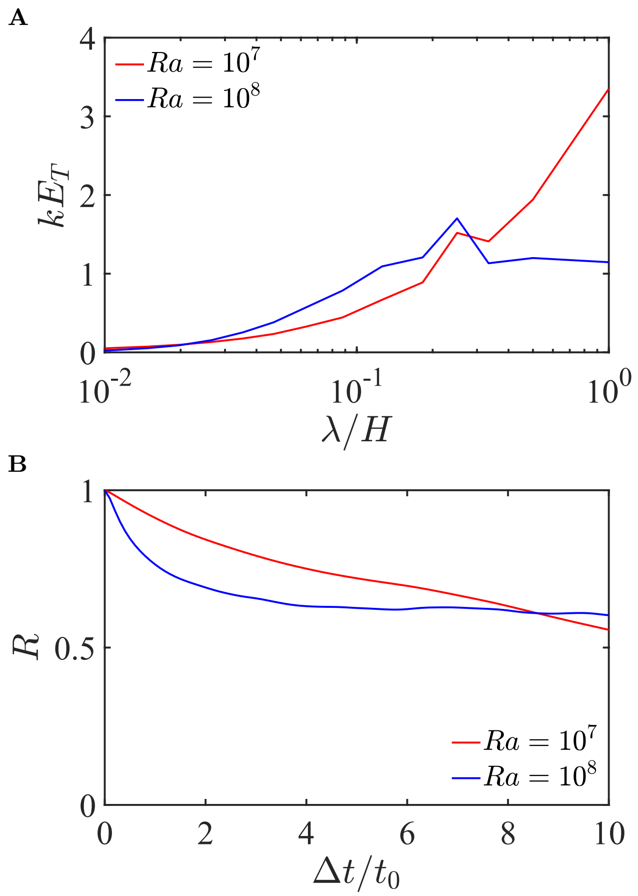

Fig. S5. The spatial energy spectra and temporal correlations of wall temperatures. (A) Premultiplied horizontal energy spectra kE_T of wall temperature outputs T'_w based on simplified control. (B) Evolution of temporal correlation R of wall temperature outputs with increasing time intervals $\triangle t$.

Table S1. Metaparameters of the deep reinforcement learning process.

Metaparameters	Value
n, the number of n-step discounted reward	5
γ, the discount factor	0.95
N, the minibatch size	64
c, the clipping range for the clipped random noise ϵ	0.3
σ_q, the standard deviation of the smooth target q	0.2
σ_0, the initial standard deviation for exploration noise	0.1
λ, the threshold value for the disturbed distance	0.03
α, the adaptive scaling factor of the disturbed distance	1.01
ξ, the mean reversion constant of the OU-process noise	0.15
μ, the mean value of the OU-process noise	0
Replay buffer memory	30000
Learning rate of the actor network	0.0001
Learning rate of the critic network	0.0002
Critic network regularization	0.0001
Soft update critic	0.001
One action duration	$0.005t_0$
One state duration	$5t_0$
One episode duration	$100t_0$
Actor update frequency	5
Target update frequency	5
Noise actor update frequency	5
Environment for DRL agent	Python, single-core serial
Environment for DNS	Fortran, MPI parallel

Table S2. Heat transfer enhancement via simplified control using edge sensor measurements

Cases	n_m	Nu	η
1E7	Full resolution	21.6	25.3%
1E7	64	21.6	25.6%
1E7	32	21.7	26.2%
1E7	16	21.7	26.0%
1E8	Full resolution	40.4	24.3%
1E8	64	40.5	24.5%
1E8	32	40.4	24.1%
1E8	16	40.3	23.9%

Table S3. Heat transfer enhancement via simplified control using temporally periodic measurements

Cases	$\triangle t_m/t_0$	Nu	η
1E7	0	23.2	35.1%
1E7	0.5	22.9	33.1%
1E7	1.0	22.6	31.4%
1E7	1.5	22.5	30.6%
1E7	2.0	21.4	30.4%
1E7	3.0	21.3	29.6%
1E7	4.0	21.2	29.2%
1E7	5.0	21.2	28.9%
1E8	0	44.8	37.8%
1E8	0.5	42.8	31.5%
1E8	1.0	42.1	29.4%
1E8	1.5	41.9	28.9%
1E8	2.0	41.8	28.5%
1E8	3.0	41.5	27.7%
1E8	4.0	41.4	27.3%
1E8	5.0	41.3	26.9%

Table S4. Heat transfer enhancement via simplified control using coarser actuators

Cases	n_c	Nu	η
1E7	Full resolution	23.2	35.1%
1E7	64	23.2	35.1%
1E7	32	22.9	33.0%
1E7	16	22.0	28.1%
1E7	8	21.1	22.8%
1E8	Full resolution	44.8	37.8%
1E8	64	43.8	34.8%
1E8	32	42.7	31.4%
1E8	16	40.6	24.8%
1E8	8	39.2	20.6%

Table S5. Heat transfer enhancement via simplified control with time delay

Cases	$\triangle t_c/t_0$	Nu	η
1E7	0	23.2	35.1%
1E7	0.5	22.8	32.3%
1E7	1.0	22.3	29.9%
1E7	1.5	22.0	28.1%
1E7	2.0	21.8	26.9%
1E7	3.0	21.8	26.7%
1E7	4.0	21.7	25.9%
1E7	5.0	21.6	25.5%
1E8	0	44.8	37.8%
1E8	0.5	42.1	29.3%
1E8	1.0	41.5	27.7%
1E8	1.5	41.1	26.3%
1E8	2.0	40.8	25.5%
1E8	3.0	40.8	25.3%
1E8	4.0	40.4	24.3%
1E8	5.0	40.4	24.1%

Table S6. Heat transfer enhancement under integrated real-world configurations

Cases	Control model	Nu	η
1E7	DRL-based	21.4	24.6%
1E7	simplified	21.0	22.3%

Movie S1. Flow field evolution ($Ra = 10^7$) with and without control.

Evolution of temperature isosurfaces for the flow in case 1E7. The left part shows the non-actuated case, while the right part represents the case after DRL-based control. Both sides start from the same initial state. Compared to the non-actuated case, the DRL-based control induces significant hot plume ejections originating from the lower wall and penetrating deep into the central flow region.

Movie S2. Flow field evolution ($Ra = 10^8$) with and without control.

Evolution of temperature isosurfaces for the flow in case 1E8. Descriptions of the flow dynamics and control effects are the same as Movie S1.

Movie S3. Flow field evolution ($Ra = 5 \times 10^8$) with and without control.

Evolution of temperature isosurfaces for the flow in case 5E8. Descriptions of the flow dynamics and control effects are the same as Movie S1.

References

1. T Lee, J Kim, C Lee, Turbulence control for drag reduction through deep reinforcement learning. *Phys. Rev. Fluids* **8**, 024604 (2023).
2. V Belus, et al., Exploiting locality and translational invariance to design effective deep reinforcement learning control of the 1-dimensional unstable falling liquid film. *AIP Adv.* **9** (2019).
3. C Vignon, J Rabault, R Vinuesa, Recent advances in applying deep reinforcement learning for flow control: Perspectives and future directions. *Phys. Fluids* **35** (2023).
4. J Rabault, A Kuhnle, Accelerating deep reinforcement learning strategies of flow control through a multi-environment approach. *Phys. Fluids* **31** (2019).
5. P Palanisamy, *TensorFlow 2 Reinforcement Learning Cookbook: Over 50 recipes to help you build, train, and deploy learning agents for real-world applications.* (Packt Publishing), (2021).
6. R Verzicco, P Orlandi, A finite-difference scheme for three-dimensional incompressible flows in cylindrical coordinates. *J. Comput. Phys.* **123**, 402–414 (1996).
7. EP Van Der Poel, R Ostilla-Mónico, J Donners, R Verzicco, A pencil distributed finite difference code for strongly turbulent wall-bounded flows. *Comput. & Fluids* **116**, 10–16 (2015).
8. X Zhu, et al., Afid-gpu: a versatile navier–stokes solver for wall-bounded turbulent flows on gpu clusters. *Comput. Phys. Commun.* **229**, 199–210 (2018).
9. P Wei, G Ahlers, Logarithmic temperature profiles in the bulk of turbulent rayleigh–bénard convection for a prandtl number of 12.3. *J. Fluid Mech.* **758**, 809–830 (2014).
10. CB Zhao, et al., Modulation of turbulent rayleigh-bénard convection under spatially harmonic heating. *Phys. Rev. E* **105**, 055107 (2022).
